# Individualizing Medicinal Therapy Post Heart Stent Implantation: Tailoring for Patient Factors

**DOI:** 10.7759/cureus.43977

**Published:** 2023-08-23

**Authors:** Tamam Mohamad, FNU Jyotsna, Umer Farooq, Aroob Fatima, Indrani Kar, Sundal Khuwaja, Unaib Ahmed Memon, Versha Kumari, Piyush Puri, Zaid M Aslam, Zachary Elder, Giustino Varrassi, Antonella Paladini, Mahima Khatri, Satesh Kumar, Muhammad Ali Muzammil

**Affiliations:** 1 Cardiology, Wayne State University, Detroit, USA; 2 Medicine, Dr. B.R. Ambedkar State Institute of Medical Sciences, Mohali, IND; 3 Medicine, CMH (Combined Military Hospital) Lahore Medical College and Institute of Dentistry, Lahore, PAK; 4 Medicine, Ejaz Sikandar Memorial Hospital, Kanganpur, PAK; 5 Medicine, Lady Hardinge Medical College, New Delhi, IND; 6 Medicine, Liaquat University of Medical and Health Sciences, Jamshoro, PAK; 7 Internal Medicine, Liaquat University of Medical and Health Sciences, Jamshoro, PAK; 8 Medicine, Adesh Institute of Medical Science and Research, Bathinda, IND; 9 Internal Medicine, Ziauddin University, Karachi, PAK; 10 Medical Education, American University of the Caribbean School of Medicine, Cupecoy, SXM; 11 Pain Medicine, Paolo Procacci Foundation, Rome, ITA; 12 Department of MESVA (Life, Health, and Environmental Sciences), University of L'Aquila, L'Aquila, ITA; 13 Medicine and Surgery, Dow University of Health Sciences, Karachi, PAK; 14 Medicine and Surgery, Shaheed Mohtarma Benazir Bhutto Medical College, Karachi, PAK; 15 Internal Medicine, Dow University of Health Sciences, Karachi, PAK

**Keywords:** drug-eluting stents, bare metal stents, coronary stent implantation, tailored treatment strategies, technological advancement, cardiovascular medicine, therapy, implantation, stent, heart

## Abstract

The field of cardiovascular medicine is undergoing a transformative shift towards personalized medicinal therapy, particularly in the context of post stent implantation. This narrative review explores the significance, challenges, and future directions of individualized treatment strategies for patients with coronary stents. The review highlights the pivotal role of personalized approaches in optimizing treatment efficacy and minimizing adverse events. Real-world clinical studies and trials underscore the importance of tailoring antiplatelet therapy based on platelet function testing, genetic testing, and risk scoring. These studies reveal that personalized medicinal treatment improves clinical outcomes by balancing preventing thrombotic events and mitigating bleeding risks. Challenges, including cost, test availability, patient adherence, and ethical considerations, are discussed in depth, shedding light on the complexities of implementing personalized approaches. Technological advancements, including omics data integration, artificial intelligence, and big data analytics, shape the future of personalized medicinal therapy. These tools enable precise pharmacogenomic selection of medications and the development of integrated risk-scoring systems. Patient engagement and education are also central, with empowered patients and remote monitoring contributing to collaborative decision-making. In conclusion, the narrative review underscores that personalized medicinal therapy post stent implantation holds immense promise for revolutionizing cardiovascular care. By embracing a comprehensive approach that considers genetics, clinical factors, and patient preferences, healthcare providers can optimize treatment outcomes and improve patient quality of life. The evolving landscape of personalized medicine offers a glimpse into a future where tailored treatment strategies become the cornerstone of precision cardiovascular care.

## Introduction and background

In recent decades, there have been notable advancements in interventional cardiology that have greatly transformed how we approach the management of coronary artery disease (CAD). One significant development has been the introduction of coronary stent implantation, which has profoundly impacted the field [1]. The utilization of percutaneous coronary intervention (PCI) along with stent placement has emerged as a fundamental approach to managing CAD, leading to enhanced short-term and long-term outcomes. Nevertheless, post-stent implantation care, particularly regarding medicinal therapy, remains an active research area. As the field of cardiovascular care continues to advance, there is a growing recognition of the importance of tailoring treatment approaches to the unique characteristics of each patient. This narrative review explores the essential task of customizing medication therapy after heart stent implantation, emphasizing adapting interventions to accommodate various patient factors [2].

The extensive adoption of coronary stent implantation has resulted in a significant decrease in acute coronary syndromes and the necessity for subsequent revascularization procedures. Based on statistics from the American Heart Association, it is estimated that approximately 600,000 coronary stent procedures are conducted each year in the United States [3]. Implementing this procedure has significantly contributed to a notable reduction in mortality rates associated with CAD. Mortality rates have observed a substantial decline of 36.4% from 2006 to 2016. This highlights the crucial significance of stent implantation in managing CAD [4]. Nevertheless, the effectiveness of coronary stenting relies on achieving an ideal equilibrium between mechanical revascularization and pharmacological treatment. According to a meta-analysis of randomized controlled trials, stent implantation effectively reduces the risk of restenosis and target vessel revascularization [5]. However, it is essential to note that the rate of major adverse cardiovascular events (MACE) remains significant, standing at 12.4% during the initial year following stent placement. This underscores the importance of implementing effective pharmaceutical treatment with mechanical intervention [6].

In addition, patient-related factors significantly influence the clinical outcomes of post-stent implantation care. Various factors, such as age, gender, comorbidities, genetics, and socioeconomic factors, play a significant role in the complex interplay that influences the pharmacokinetics and pharmacodynamics of medications [7]. The 2018 European Heart Rhythm Association Practical Guide on the use of non-vitamin K antagonist oral anticoagulants in patients with atrial fibrillation revealed that elderly patients aged 75 years and above demonstrated modified reactions to antiplatelet medicines such as clopidogrel [8]. As a result, it is imperative to administer adjusted dosages to minimize the potential risks of bleeding. Similarly, it is worth noting that genetic polymorphisms have the potential to exert a substantial influence on the metabolism of clopidogrel. This, in turn, may result in treatment resistance within specific populations. The high occurrence of diabetes mellitus among patients with CAD emphasizes the importance of personalization [9]. Diabetes is estimated to affect approximately 34.2 million individuals in the United States [10]. It has been observed that individuals with diabetes have a higher likelihood of needing coronary intervention. A systematic review and meta-analysis by Hwang et al. revealed that diabetic patients who underwent stent implantation had a higher incidence of MACE than non-diabetic individuals [11]. This situation prompts inquiries regarding the customization of antidiabetic and antithrombotic treatment plans for this specific group of patients [11,12].

Given the ongoing significance of coronary stent implantation in managing CAD, it is crucial to prioritize the optimization of medicinal therapy following stent placement [11]. The mechanical advantages of stenting are apparent; however, the high incidence of MACE calls for a personalized strategy that considers factors specific to each patient. This narrative review examines the diverse aspects of medicinal therapy after stent placement, focusing on the intricate considerations associated with age, gender, genetics, comorbidities, and socioeconomic factors. This review seeks to enhance treatment strategies in precision cardiovascular medicine by examining the relationship between patient characteristics and pharmacological interventions. The goal is to contribute to refining these strategies to improve patient outcomes.

## Review

Types of stents and medicinal therapy in CAD management

CAD is recognized as a significant contributor to morbidity and mortality on a global scale. Within the field of interventional cardiology, implanting coronary stents has become a crucial procedure for restoring blood flow and managing the complications associated with CAD [13]. This review explores various coronary stents, such as bare metal stents (BMS) and drug-eluting stents (DES). It highlights the critical role of medicinal therapy after stent implantation. The primary emphasis is placed on using antiplatelet agents, including aspirin and P2Y12 inhibitors, and potential complementary therapies.

Various Types of Coronary Stents

BMS is one of the initial stent designs. These stents are composed of a metal scaffold deployed to preserve the patency of the coronary artery following balloon angioplasty. Although BMSs have demonstrated efficacy in mitigating acute vessel closure, they are constrained by the potential for restenosis, which involves the undesirable occurrence of excessive tissue growth resulting in the re-narrowing of the vessel [14].

DES was developed as a response to the limitations observed in BMS, aiming to mitigate the restenosis problem. These stents are coated with pharmacological agents that effectively inhibit cell proliferation, thereby significantly reducing the probability of restenosis. The drug classes most frequently utilized for coating purposes encompass antiproliferative agents such as sirolimus and paclitaxel [15]. Additionally, there has been a recent emergence of newer agents specifically engineered to possess improved safety profiles. DESs have had a profound impact on the field of coronary intervention, revolutionizing the way we approach and treat CAD. According to a study by Meraj et al., it was found that DES demonstrated a nearly 50% reduction in the risk of target lesion revascularization compared to BMS [16]. This significant improvement can be primarily attributed to the inhibitory effect of DES on neointimal hyperplasia. As a result, DES has emerged as the prevailing standard of care for most patients undergoing coronary stent implantation [15,17].

Medicinal Therapy in the Context of Coronary Stents

The administration of medicinal therapy following stent implantation is of utmost importance in preventing stent thrombosis, a potentially severe complication. Stent thrombosis is closely linked to significant mortality rates, underscoring the crucial significance of antiplatelet therapy [17]. The main emphasis of post-stent medical therapy centers on antiplatelet agents, which effectively hinder platelet aggregation and mitigate the likelihood of thrombus formation within the stent. Acetylsalicylic acid (commonly known as aspirin) is widely recognized as a fundamental component of antiplatelet therapy following the implantation of a stent. It permanently hinders the activity of the cyclooxygenase (COX) enzyme, which plays a critical role in platelet aggregation and the synthesis of thromboxane A2. Numerous clinical trials have consistently shown the effectiveness of aspirin in mitigating adverse cardiovascular events [18]. According to the guidelines set forth by the American College of Cardiology (ACC) and the American Heart Association (AHA), it is recommended that aspirin be prescribed as a long-term therapy following the implantation of a stent [17].

P2Y12 inhibitors belong to a class of antiplatelet agents targeting the ADP receptor found on platelets. By doing so, these inhibitors effectively hinder the activation and aggregation of platelets. Frequently utilized P2Y12 inhibitors comprise clopidogrel, prasugrel, and ticagrelor. Clopidogrel is a prodrug that undergoes hepatic metabolism to its active form. It is widely utilized in clinical practice owing to its cost-effectiveness and widespread availability [18,19]. However, the effectiveness of the treatment may be influenced by genetic variations in metabolism, resulting in variability in individual responses. Prasugrel is a recently developed P2Y12 inhibitor that has been found to provide more robust and consistent platelet inhibition when compared to clopidogrel. The treatment has shown significant advantages in patients with a high risk of cardiovascular complications. However, it is essential to exercise caution due to the potential for an elevated risk of bleeding. Ticagrelor is distinguished from clopidogrel and prasugrel by its characteristic as a reversible P2Y12 inhibitor [19]. This medication offers reliable platelet inhibition and has demonstrated mortality benefits in individuals diagnosed with acute coronary syndromes. P2Y12 inhibitors are commonly prescribed as part of dual antiplatelet therapy (DAPT) regimens, in conjunction with aspirin, for varying durations based on the patient's clinical characteristics and the type of stent utilized. The duration of DAPT has been the focus of research, and recent studies indicate that shorter durations of DAPT may be suitable for specific patients to reduce the risk of bleeding without compromising effectiveness[19].

Possible Adjunctive Therapies

In addition to antiplatelet agents, researchers are further investigating other adjunctive therapies to optimize coronary stent implantation outcomes.

Anticoagulants: Emerging anticoagulant medications such as rivaroxaban and apixaban, initially designed for treating atrial fibrillation, are currently under investigation for their potential use in combination with antiplatelet therapy. The aim is to effectively decrease the occurrence of ischemic events while minimizing the associated bleeding risks. Proton pump inhibitors (PPIs) are occasionally prescribed to mitigate the potential occurrence of gastrointestinal bleeding that may be linked to antiplatelet therapy [20]. Nevertheless, the utilization of these substances may affect the absorption of antiplatelet agents, thus necessitating meticulous deliberation.

Genetic testing: The utilization of genetic testing to customize antiplatelet therapy to predict an individual's response to these medications is a developing concept that shows potential for enhancing treatment outcomes. Implementing coronary stents has significantly transformed the management of CAD, with the selection of the appropriate stent type playing a crucial role in determining patient outcomes. However, it is essential to note that the sole use of stent implantation may not be adequate to achieve optimal results [21]. Pharmacological therapy, specifically using antiplatelet agents such as aspirin and P2Y12 inhibitors, is of utmost importance in preventing complications such as stent thrombosis. As the field of medicine continues to advance, there is a growing recognition of the importance of tailoring medicinal therapy to individual patients. By taking into account patient-specific factors and considering potential adjunctive treatments, we can enhance the effectiveness of coronary stent implantation in the era of precision cardiovascular medicine [22].

Patient Factors Affecting Medicinal Therapy

The process of optimizing medicinal therapy following coronary stent implantation is a multifaceted endeavor that requires a comprehensive comprehension of individual patient factors. These factors encompass a diverse range of variables, including age, gender, comorbidities, allergies, genetic predispositions, co-medications, and lifestyle choices. This article explores the complex relationship between patient-related factors and their impact on the selection, dosage, and effectiveness of medications in the context of coronary stent implantation [23].

Age and gender: The age and gender of individuals play a crucial role in influencing the pharmacokinetics and pharmacodynamics of medications administered after stent implantation. Elderly individuals frequently demonstrate modified reactions to medications as a result of age-associated alterations in drug metabolism and elimination [24]. For example, diminished renal and hepatic function may result in extended drug exposure, thereby elevating the likelihood of experiencing adverse events. Research has indicated that elderly individuals may experience a reduced antiplatelet response when using antiplatelet agents such as clopidogrel. This highlights the importance of customized dosing strategies to ensure sufficient platelet inhibition. In addition, it is worth noting that elderly patients may exhibit a higher vulnerability to experiencing bleeding events. Hence, achieving a delicate equilibrium between the effectiveness of antithrombotic measures and the potential for bleeding complications is crucial. Implementing modified dosages of antiplatelet agents and conducting diligent monitoring of bleeding parameters are effective strategies to achieve optimal outcomes in this specific population [25]. The recognition of gender-based differences in drug responses is growing. Research findings indicate that there may be variations in platelet responses to antiplatelet agents between women and men. Various factors, including hormonal influences, genetic variations, and variations in body composition, contribute to these disparities. Considering gender-specific characteristics when tailoring medication doses is essential in enhancing therapeutic outcomes [26].

Comorbidities: The presence of comorbid conditions such as diabetes, hypertension, and renal dysfunction significantly influences the selection of medications and their efficacy. The prevalence of diabetes among patients with CAD is significant, necessitating a comprehensive therapeutic approach for these individuals [27]. The presence of diabetes has the potential to negatively impact platelet function, which may require the implementation of more potent antiplatelet treatment protocols. Moreover, individuals diagnosed with diabetes are more susceptible to experiencing renal dysfunction, which can have an impact on the clearance of medications. Novel pharmaceuticals for the treatment of diabetes, such as sodium-glucose cotransporter-2 (SGLT2) inhibitors, have exhibited positive effects on cardiovascular health. As a result, these medications can potentially impact the choice of treatment options within this specific patient population [28].

Hypertension, often found alongside CAD, necessitates using antihypertensive medications for effective management. It is essential to consider the potential drug interactions between antiplatelet agents and specific antihypertensives, such as angiotensin-converting enzyme (ACE) inhibitors, to prevent any reduction in the effectiveness of antiplatelet therapy. Impaired renal function can have a negative impact on the elimination of drugs, which may result in drug accumulation and an elevated risk of experiencing adverse effects [29]. It is imperative to make dosing adjustments for medications cleared by the kidneys, such as P2Y12 inhibitors and anticoagulants, to avoid potential toxicity.

Allergies and intolerances: Considering patient allergies and medication intolerances is paramount when determining the appropriate medication [30]. For example, individuals with hypersensitivity to aspirin may necessitate using alternative antiplatelet agents such as clopidogrel or ticagrelor. The top priority is ensuring patient safety while maximizing the therapy's effectiveness.

Genetic factors: Genetic polymorphisms have the potential to significantly impact drug metabolism and response, thereby contributing to the variability observed in treatment outcomes. The CYP2C19 polymorphism has been found to have an effect on the metabolic activity of clopidogrel [31]. Individuals with reduced function alleles demonstrate a decrease in the conversion of clopidogrel into its active form. This could result in a reduction in the effectiveness of the antiplatelet properties of the medication. The utilization of genetic testing to guide antiplatelet selection and dosing has garnered significant attention, especially within high-risk populations [32].

Co-medications: The simultaneous use of multiple medications can result in drug interactions that can potentially modify therapeutic agents' effectiveness and safety characteristics. PPIs are frequently prescribed to reduce the risk of gastrointestinal bleeding that can occur with antiplatelet therapy [33]. It is important to note that the use of PPIs can affect the absorption of clopidogrel. It may be necessary to consider alternative antiplatelet agents or make adjustments to the dose of PPIs.

Impact of Smoking and Lifestyle Factors

The efficacy of medications and patient adherence can be influenced by lifestyle choices, specifically smoking and dietary habits. Smoking has been found to negatively affect platelet function and the metabolism of certain medications, which may lead to a potential decrease in their effectiveness. In addition, it is worth noting that patients who engage in smoking may necessitate higher dosages of antiplatelet agents in order to attain equivalent levels of platelet inhibition. The consumption of alcohol and dietary components has the potential to interact with medications [34]. For example, the consumption of grapefruit juice has the potential to impede the activity of drug metabolism enzymes, thereby influencing the concentrations of certain drugs within the body. It is imperative for healthcare providers to provide patients with education regarding these interactions and offer guidance on making suitable dietary choices. Various lifestyle factors can influence the adherence to prescribed medications by patients. Non-compliance with prescribed treatment regimens undermines the efficacy of the treatment and exposes patients to increased risks of negative outcomes [35].

Optimizing medicinal therapy following coronary stent implantation necessitates a patient-centered approach that considers various factors. Age, gender, comorbidities, allergies, genetic variations, co-medications, smoking, and lifestyle choices influence the therapeutic landscape [36]. Healthcare providers must thoroughly evaluate these factors to customize treatment plans, choose suitable medications, establish the most effective dosages, and minimize potential interactions. In the context of precision medicine, it is crucial to acknowledge and effectively manage the complex relationship between patient-specific factors. This is necessary in order to improve outcomes and guarantee the safety and effectiveness of medicinal therapy following stent placement [36,37]. 

Antiplatelet Therapy Duration and DAPT

The optimal duration of DAPT after coronary stent implantation has been extensively studied and discussed. This determination considers factors such as the type of stent used, the characteristics of the patient, and the delicate balance between the risks of ischemia and bleeding [38]. Although guidelines offer general recommendations, it is crucial to emphasize the significance of customizing the duration of DAPT based on the specific risk profiles of individual patients.

**Figure 1:**
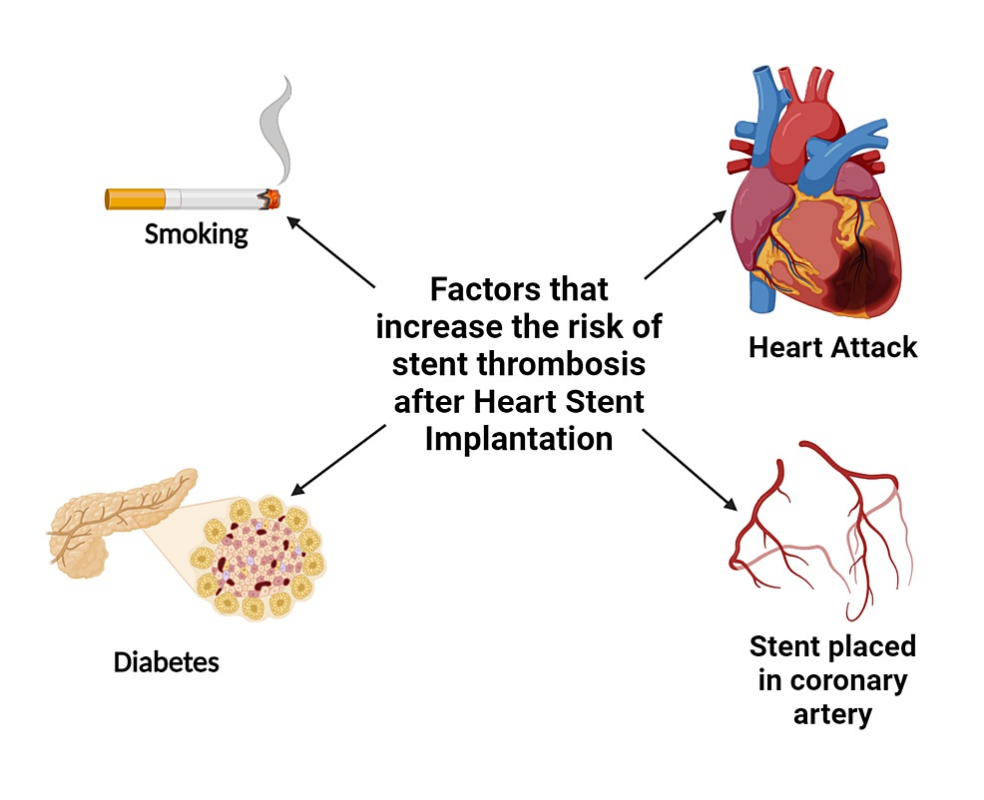
Range of factors correlated with in-stent thrombosis Credit: Authors

Stent selection and patient characteristics: The decision regarding the use of BMS or DES is a critical factor in determining the duration of DAPT. DESs, which incorporate polymer coatings that release antiproliferative drugs, have demonstrated a notable reduction in restenosis rates when compared to BMSs [38,39]. Therefore, recipients of DESs often require extended durations of DAPT to minimize the potential risk of late stent thrombosis. The introduction of newer-generation DESs with biocompatible polymers has led to a trend towards shorter durations of DAPT, while maintaining safety standards. Figure 1 illustrates various elements linked with immediate thrombosis.

Patient characteristics can further influence the duration of DAPT decisions. Various factors such as age, gender, comorbidities, and genetic predispositions significantly affect the risks of thrombosis and bleeding. Patients who are elderly or have a history of bleeding may require shorter durations of DAPT to minimize bleeding risks. On the other hand, patients with diabetes or those with complex CAD may necessitate longer durations of DAPT to ensure sufficient platelet inhibition [40]. Customizing DAPT duration based on these characteristics enhances both safety and efficacy outcomes.

The recommendations for DAPT duration have undergone significant evolution. Historically, conventional practices have recommended a uniform approach of 12 months of DAPT after DES implantation [41]. Nevertheless, the accumulation of evidence and clinical trials have challenged this notion. Studies such as the PRODIGY trial have shown that shorter durations of DAPT, precisely six months, in patients who have received newer-generation DES do not pose a safety risk. These findings have led to the development of personalized DAPT duration [42].

On the other hand, in patients at high risk, there may be potential advantages to extending the duration of DAPT. The DAPT study has provided evidence that prolonging DAPT beyond the standard 12-month duration can effectively decrease the occurrence of MACE in specific populations [43]. However, it is essential to note that this benefit comes with the trade-off of an increased risk of bleeding. Therefore, the transition towards personalized approaches to DAPT is driven by a thorough evaluation of ischemic and bleeding risks in every patient.

Achieving a balance between ischemic and bleeding risks requires a thorough assessment of the optimal duration for DAPT. The administration of prolonged DAPT has decreased the likelihood of thrombotic events while concurrently increasing the potential for bleeding complications. On the contrary, a shorter duration of DAPT reduces the possibility of bleeding events [44]. However, it may also elevate the risk of stent thrombosis. The attainment of equilibrium between these contrasting risks is fundamental to implementing personalized DAPT strategies.

The utilization of risk scores has become increasingly significant in informing decisions regarding the duration of DAPT. Assessment tools such as DAPT Risk Calculator (American College of Cardiology, Washington, D.C., United States), PRECISE-DAPT, and ARC-HBR (European Cardiovascular Research Center (CERC), Essonne, France) are utilized to categorize patients according to their unique risk profiles [45]. The scores incorporate clinical variables, stent type, and bleeding risk factors to guide the appropriate duration of DAPT. Nevertheless, it is essential to acknowledge that no scoring system can guarantee absolute accuracy, and it is crucial to prioritize clinical judgment above all else.

Interdisciplinary collaboration becomes essential in patients at a high risk of experiencing both ischemic and bleeding events. It is imperative for cardiologists, hematologists, and gastroenterologists to collaboratively evaluate the potential risks and benefits associated with the duration of DAPT [46]. The implementation of shared decision-making between healthcare providers and patients is crucial to effectively incorporate each patient's unique preferences and values into the decision-making process regarding their treatment options.

Within the context of coronary stent implantation, the duration of DAPT plays a crucial role in determining patient outcomes. The decision-making process has evolved from strict guidelines to personalized approaches, considering factors such as stent type, patient characteristics, and detailed risk assessments [47]. The evolving body of evidence has led to a shift in the approach to the duration of DAPT, moving away from a standardized requirement to a more personalized strategy. This new approach considers carefully assessing the balance between the risks of ischemic events and bleeding complications. Although risk scores provide valuable guidance, it is essential to recognize that clinical expertise and patient preferences play a crucial role [44-47]. The future of DAPT duration depends on ongoing research, further development of risk assessments, and the adoption of a patient-centered approach that prioritizes both effectiveness and safety in precision cardiovascular care.

Emerging biomarkers and imaging techniques

Recent developments in cardiovascular medicine have prompted the investigation of new biomarkers and imaging techniques for tailoring medicinal therapy after the implantation of coronary stents [44]. The dynamic nature of this field strives to improve treatment accuracy by identifying individual patient factors that impact the response to therapy and prognosis. Platelet function testing, genetic testing, and non-invasive imaging techniques have emerged as promising strategies for customizing medicinal treatment and transforming the approach to post-stent care [47].

Platelet function testing is a crucial component in evaluating the effectiveness of antiplatelet therapy and customizing it based on individual patient responses. While traditional assays such as aggregometry provide valuable information on platelet aggregation, more recent methods offer improved granularity [45-47]. The VerifyNow P2Y12 and Multiplate® (F. Hoffmann-La Roche AG, Basel, Switzerland) assays are point-of-care tests that offer prompt evaluation of platelet function, enabling timely modification of antiplatelet therapy [48]. They provide valuable information regarding the efficacy of medications such as aspirin and P2Y12 inhibitors, facilitating personalized dosage modifications based on platelet reactivity [42].

The vasodilator-stimulated phosphoprotein (VASP) assay is a method used for measuring the activity of VASP. This flow cytometric technique is utilized to measure platelet activation markers. It holds significant value in the assessment of clopidogrel responsiveness [49]. The test detects elevated levels of platelet reactivity during treatment, leading to modifications in the antiplatelet medication plan. Platelet function testing enables healthcare professionals to customize the dosage and choice of antiplatelet therapy for patients [43]. Elevated platelet reactivity may necessitate an increase in dosage or a transition to more potent medications. On the other hand, decreased platelet reactivity could be utilized to determine the need for dosage reduction to minimize the potential for bleeding complications. The personalized approach in this context aims to maximize therapeutic outcomes while minimizing adverse events.

Genetic Testing

Genetic testing is an important tool for personalized medicinal therapy as it allows the examination of genetic variations that intricately influence drug metabolism and response. Genetic polymorphisms influence the metabolism of clopidogrel in the *CYP2C19* gene. Patients who have alleles with reduced function demonstrate a decreased response to clopidogrel [17]. As a result, it becomes necessary to consider alternative P2Y12 inhibitors such as prasugrel or ticagrelor. Genetic testing enables the customization of antiplatelet selection, thereby mitigating the potential for treatment resistance.

Pharmacogenomics

Besides *CYP2C19*, genetic testing can provide insights into variations in drug metabolism enzymes and transporters, which can impact the effectiveness and safety of medications. Customizing the selection of antiplatelet and anticoagulant medications according to genetic profiles is essential for achieving the most favorable treatment results. Non-invasive imaging modalities are crucial in providing valuable information about coronary anatomy, physiology, and plaque vulnerability, thereby assisting in making informed therapeutic decisions [18]. Intravascular ultrasound (IVUS) and optical coherence tomography (OCT) are two imaging techniques that offer high-resolution visuals of coronary arteries. These technologies play a crucial role in optimizing stents and evaluating stent apposition. OCT provides valuable information about the coverage of stent struts, which assists in determining the appropriate duration of DAPT. Fractional flow reserve and instantaneous wave-free ratio are physiological indices assessing coronary stenosis's functional significance [19-21]**.** These factors play a crucial role in determining revascularization strategies, which may impact the necessity of stent placement and the duration of DAPT. CT angiography (CTA) allows for the non-invasive evaluation of coronary anatomy and plaque characteristics, providing valuable information regarding the feasibility of stenting and post-stent monitoring [21].

Tailoring Medicinal Therapy

Non-invasive imaging modalities play a crucial role in guiding stent implantation strategies, thereby improving the overall outcomes of stent procedures. The optimization of stent deployment has a significant impact on the subsequent antiplatelet regimen and the duration of DAPT. Moreover, using imaging techniques to gain insights into the vulnerability of plaque can provide valuable guidance for implementing aggressive lipid-lowering treatments and anti-inflammatory interventions. Incorporating innovative biomarkers and imaging modalities in the customization of medical therapy following coronary stent implantation shows excellent potential [23-25]. Platelet function testing enables clinicians to personalize antiplatelet regimens by utilizing real-time platelet reactivity data. Genetic testing enables the personalized selection of antiplatelet and anticoagulant agents, thereby addressing treatment resistance and enhancing safety measures. Non-invasive imaging techniques offer valuable insights into the deployment of stents and plaque vulnerability, informing decisions regarding stent strategy and subsequent medicinal therapy. These advancements collectively contribute to the era of precision cardiovascular medicine, enhancing patient outcomes by implementing personalized therapeutic approaches [27].

Role of platelet reactivity testing

Platelet function testing is a vital aspect of cardiovascular medicine as it plays a crucial role in evaluating the specific responses of individual patients to antiplatelet therapy [31]. This innovative tool offers valuable insights into platelet reactivity, enabling healthcare professionals to make informed decisions regarding treatment adjustments and ultimately impacting patient outcomes. The value of platelet function testing resides in its capacity to individualize antiplatelet therapy, enhance treatment effectiveness, and reduce the likelihood of unfavorable effects [32].

Significance of Platelet Function Testing

Evaluation of response variability: The variability in platelet response to antiplatelet agents is substantial across patients, primarily attributed to genetic predispositions, comorbidities, and drug interactions. Platelet function testing, utilizing techniques such as aggregometry, VerifyNow P2Y12, and Multiplate assays enable measuring platelet reactivity and identifying patients exhibiting suboptimal antiplatelet response [49]. This information is essential for comprehending the efficacy of prescribed therapies.

Tailoring medication selection: Various antiplatelet agents elicit their effects via unique mechanisms. Platelet function testing is crucial in selecting the most suitable antiplatelet agent, considering patients' responses [4-6]. An instance where a subpar reaction to clopidogrel is observed necessitates the evaluation of alternative agents, such as prasugrel or ticagrelor, to attain the most effective platelet inhibition. The optimization of dosages can be achieved through platelet function testing, which enables accurate adjustments to be made. Clinicians can optimize antiplatelet dosages to achieve the desired level of platelet inhibition by assessing platelet reactivity. This customization approach effectively addresses under- and over-inhibition, enhancing treatment effectiveness and decreasing bleeding risks [9,11,12].

Effect of Treatment Modification

Tailored antiplatelet regimens: The utilization of platelet function testing enables healthcare professionals to make adjustments to antiplatelet regimens according to the specific responses of individual patients. We should consider switching patients identified as poor responders to more potent antiplatelet agents to ensure sufficient platelet inhibition [28]. On the other hand, individuals at an increased risk of bleeding may have their medication dosages adjusted to achieve a proper equilibrium between the occurrence of ischemic events and bleeding complications.

The mitigation of treatment resistance: Early identification of patients exhibiting a diminished response to antiplatelet therapy prevents the development of treatment resistance [29]. Modifying antiplatelet therapy per the results of platelet function testing mitigates the potential for stent thrombosis and recurrent ischemic events, thereby optimizing treatment efficacy.

Impact on Clinical Outcomes

The primary objective of antiplatelet therapy is to mitigate the occurrence of stent thrombosis and subsequent ischemic events. Platelet function testing is a crucial procedure that aims to verify that patients attain the intended degree of platelet inhibition, thereby mitigating the likelihood of thrombotic complications [30]. This results in enhanced clinical outcomes characterized by reduced occurrences of myocardial infarction, target lesion revascularization, and overall cardiovascular events [31]. When considering the prevention of ischemic events, it is crucial even to consider the potential risks of bleeding complications. Platelet function testing is valuable in optimizing antiplatelet therapy to achieve the ideal level of platelet inhibition while minimizing the potential for bleeding complications. This approach aims to optimize patients' therapeutic benefits while minimizing potential harm [33-35].

The assessment of platelet function has become a fundamental aspect in customizing antiplatelet therapy, significantly transforming the approach to cardiovascular healthcare. This dynamic tool allows for the customization of treatment regimens to achieve optimal platelet inhibition by evaluating individual patient responses to antiplatelet agents [47]. The significance of platelet function testing is apparent in its contribution to treatment modification, mitigation of treatment resistance, and influence on clinical outcomes.

Real-world clinical studies and clinical trials

Cardiovascular medicine has experienced a significant transformation toward personalized medicinal therapy. Valuable insights from real-world clinical studies have influenced this shift and meticulously planned clinical trials. These studies present compelling evidence regarding the significance of customizing therapeutic approaches for individual patients, demonstrating improved outcomes and enhanced treatment effectiveness [47]. Let us explore several prominent real-world studies and clinical trials highlighting the importance of personalized medicinal therapy in cardiovascular care.

Real-World Clinical Studies

The Prevention of Bleeding in Patients with Atrial Fibrillation (AF) Undergoing Percutaneous Coronary Intervention (PCI) (PIONEER-AF-PCI) Trial investigated the implementation of individualized antithrombotic therapy for patients with AF undergoing PCI. The study compared different treatment regimens that combined antiplatelet and anticoagulant agents [44]. The objective was to identify the most effective approach that balanced the risks of thrombosis and bleeding. The findings underscore the significance of customizing therapy according to individual patient profiles to strike a careful equilibrium between ischemic and bleeding complications [44]. The RE-DUAL PCI (Randomized Evaluation of Dual Antithrombotic Therapy with Dabigatran versus Triple Therapy with Warfarin in Patients with Nonvalvular AF Undergoing PCI) Trial investigated the efficacy of dual antithrombotic therapy with dabigatran in patients with AF who required PCI [24]. The study findings indicate that implementing a personalized approach to antithrombotic therapy, taking into account patient risk factors and the type of stent used, decreased bleeding events without compromising the treatment's effectiveness.

Validation study of the DAPT score: The DAPT score, developed based on clinical trials, underwent validation in real-world populations to assess its ability to predict the most suitable duration of DAPT after stent implantation [19]. The practical implementation of risk scoring in this particular context highlighted the significance of tailoring the course of DAPT to mitigate the occurrence of unfavorable outcomes [22].

List of Pertinent Clinical Trials

TheTROPICAL-ACS (Testing Responsiveness To Platelet Inhibition On Chronic Antiplatelet Treatment For Acute Coronary Syndromes) Trial examined the customization of P2Y12 inhibitor therapy in patients diagnosed with acute coronary syndrome (ACS) undergoing PCI. The trial compared tailored therapy utilizing platelet function testing and conventional medicine [9]. The findings indicated that implementing treatment modification based on platelet function testing decreased adverse cardiovascular events. The POPular Genetics trial investigated the utilization of genetic testing to customize antiplatelet therapy in ST-segment elevation myocardial infarction patients to study patient outcome after primary PCI (POP). The results highlighted the potential advantages of genetic testing in improving the effectiveness of treatment [12].

The TAILOR-PCI (Tailored Antiplatelet Initiation to Lessen Outcomes Due to Decreased Clopidogrel Response after Percutaneous Coronary Intervention) Trial investigated the impact of genetic testing on the selection of antiplatelet therapy in patients with stable coronary artery disease who were undergoing PCI. The patients were allocated to either the standard or genotype-guided therapy group [13]. The trial highlighted the importance of genotype-guided treatment optimization, which was associated with a reduced incidence of cardiovascular events.

Summary of findings

The findings derived from clinical studies and clinical trials consistently underscore the crucial significance of personalized medicinal therapy in cardiovascular care [33]. These studies collectively highlight several important points.

Personalized Medicinal Therapy

The foundation of personalized medicinal therapy relies on conducting individualized risk assessments. Various factors, including genetic variations, comorbidities, stent type, and patient characteristics, are crucial in informing treatment decisions [35]. Achieving a balance between minimizing thrombotic events and managing bleeding risks is critical, as highlighted by real-world studies and clinical trials. Tailored therapy endeavors to optimize outcomes by addressing the intricate balance at play.

Optimization of treatment: The utilization of personalized therapy optimization, which is based on platelet function testing, genetic testing, or risk scoring, is correlated with enhanced clinical outcomes [36]. Customizing therapeutic regimens based on individual patient profiles improves the effectiveness and safety of treatment. The success observed in these studies and trials highlights the potential impact of precision medicine in cardiovascular care. The customization of treatments for individual patients, as opposed to using a standardized approach, shows potential for significantly improving patient outcomes. The collective body of real-world clinical studies and clinical trials highlights the significant influence of personalized medicinal therapy in cardiovascular care. These investigations demonstrate that personalized approaches, informed by factors such as genetics, platelet function, and clinical risk profiles, have a substantial impact on the effectiveness of treatment and the outcomes experienced by patients [38]. As cardiovascular medicine increasingly adopts precision medicine, these findings provide evidence of the potential for customized therapeutic approaches to significantly impact patient care and improve the quality of life for individuals with cardiovascular diseases [39].

Challenges and Considerations

The implementation of personalized medicinal therapy presents various challenges and considerations, despite its potential to significantly transform patient care. Multiple factors, including economic constraints and ethical dilemmas, must be carefully considered to ensure that customized treatment strategies result in enhanced clinical outcomes. It is imperative to address the challenges associated with cost, test availability, patient adherence, and ethical considerations to fully leverage personalized medicine's potential.

Expenses for diagnostic testing, other healthcare costs, and economic impact: The implementation of personalized medicinal therapy often necessitates using specialized diagnostic tests, such as genetic testing and platelet function assays. The cost of these tests can be significant, potentially strain healthcare systems and impose financial burdens on patients, especially in regions with limited resources [21]. Although personalized treatments can result in improved outcomes and decreased complications, higher initial expenses may be associated with tailored approaches. This may result in a financial obstacle, particularly in cases where insurance coverage is restricted or unavailable [23]. Integrating personalized medicine into standard practice for healthcare systems may necessitate substantial investments in infrastructure, training, and research [23]. The task of weighing the long-term advantages against immediate expenses presents a complex challenge that requires thoughtful deliberation.

Test availability and complexity of testing: There are significant disparities in accessing advanced diagnostic tests globally, with certain regions facing challenges due to inadequate facilities and limited expertise [25]. This inequality has the potential to result in disparities in patient care, thereby restricting the widespread adoption of personalized medicinal therapy. Certain diagnostic tests, particularly those involving genetic profiling, necessitate the utilization of specialized laboratories and personnel with specific training [27]. The intricate nature of this complexity may pose a challenge to achieving widespread adoption, as not all healthcare facilities may possess the requisite resources.

Patient adherence and education: Customized therapies may encompass intricate treatment regimens comprising multiple medications and subsequent follow-up appointments [26]. Ensuring patient adherence is of utmost importance, as failure to comply with prescribed treatments can compromise the effectiveness of personalized approaches. To adhere to them wholeheartedly, patients must comprehend the underlying reasoning behind personalized treatment plans [28]. Establishing precise and efficient communication channels between healthcare providers and patients is of utmost importance to promote patient adherence and successfully attain the desired outcomes.

Ethical considerations and equitable distribution: The process of personalized medicinal therapy entails evaluating the specific risk factors of each patient and customizing treatments accordingly. Ethical considerations come into play when attempting to balance the potential advantages of optimized therapy and the inherent risks associated with complex treatments. In the context of informed consent, it is essential to acknowledge that genetic testing has the potential to provide valuable insights into a patient's health that may extend beyond the specific condition being addressed [45]. Ethical dilemmas arise when considering the appropriate level of information disclosure, balancing the principles of patient autonomy and the need to protect their emotional well-being. Personalized medicine heavily relies on patient data, which has raised significant concerns regarding data privacy, ownership, and potential misuse. It is crucial to prioritize the secure storage and responsible utilization of sensitive patient information. The implementation of personalized medicinal therapy should prioritize fairness, ensuring that individuals from all socioeconomic and demographic backgrounds have equal access to advanced treatments. Ethical frameworks must be employed to inform decision-making processes to mitigate the exacerbation of pre-existing healthcare disparities [13].

Challenges in Clinical Trials and Evidence

Clinical trial design: The execution of clinical trials for personalized approaches can present inherent complexities, necessitating the consideration of factors such as patient heterogeneity and treatment variability. The suitability of traditional randomized controlled trials for evaluating tailored treatments may be limited in some instances.

Insufficient evidence: Although the potential advantages of personalized medicinal therapy are apparent, there currently needs to be more substantial evidence regarding its effectiveness in different patient populations and medical conditions. There needs to be more availability of comprehensive data to ensure widespread adoption.

Specialized training: Healthcare professionals require specialized training to interpret and apply diagnostic test results effectively. It is imperative to incorporate this education into medical curricula and ongoing professional development to ensure smooth and effective implementation.

Future directions

The field of personalized medicinal therapy following stent implantation has experienced significant advancements and continues to witness ongoing innovations that have the potential to transform cardiovascular care. As we consider the future, combining technological advances, comprehensive data integration, and sophisticated patient profiling can introduce a new era of precision medicine [39]. This approach aims to enhance patient outcomes and revolutionize the standard of care. The upcoming generation is defined by the customization of therapeutic strategies for individual patients, utilizing various tools to navigate complexity and guarantee the highest level of treatment effectiveness.

Technological Advancements

Integrating genomics, proteomics, and metabolomics holds excellent potential in elucidating the complex genetic and molecular mechanisms underlying cardiovascular diseases. As our comprehension of the genetic underpinnings of diseases advances, there will be a rise in the development of targeted therapies that specifically target molecular abnormalities. This will result in the implementation of more efficient treatment approaches [25-29]. Artificial intelligence (AI) and machine learning (ML) technologies possess the capability to analyze extensive volumes of patient data, enabling the identification of patterns and the prediction of treatment responses. By utilizing these technologies, medical professionals can make informed decisions based on data, thereby optimizing the selection of therapies for individual patients. Integrating large-scale data and real-world evidence presents an opportunity to gain comprehensive insights into treatment efficacy across diverse patient populations [33-37]. A more holistic understanding can be achieved by combining clinical trial outcomes with the accumulation of real-world patient data. Using big data analytics will empower clinicians to enhance treatment strategies and customize them according to individual patient profiles.

Customizing Treatments

Integrating genetic testing into treatment decision-making will play a crucial role in pharmacogenomics. This approach will provide valuable insights for healthcare professionals, aiding in selecting appropriate medications and dosages for patients. Utilizing pharmacogenomic insights will guarantee that patients are provided with the most efficient and secure therapeutic regimens tailored to their genetic composition [38-41]. Advanced risk scoring systems will incorporate a comprehensive array of patient characteristics, encompassing genetic predispositions as well as clinical factors. The scores provided will offer a thorough evaluation of individual risk profiles, aiding in customizing treatment plans.

Patient Engagement and Education

The concept of empowered patients involves patients taking on a more proactive role in making decisions regarding their treatment. This is achieved by equipping them with a comprehensive understanding of their disease and the various treatment options. Implementing improved patient education and engagement strategies will facilitate a collaborative decision-making process that aligns with the patient's preferences [43-45].

Remote patient monitoring: The utilization of wearable devices and telemedicine will effectively facilitate the monitoring of patients from a remote location. This will enable healthcare providers to closely track the responses to treatment and promptly intervene if necessary. The implementation of continuous monitoring will enhance the level of personalization in patient care. The implementation of personalized medicinal therapy following stent implantation has resulted in a significant transformation in cardiovascular healthcare [44-47]. The consideration of patient characteristics and the understanding of platelet reactivity guide treatment strategies toward precision and effectiveness.

Collaborative patient-centric care: The future of personalized medicinal therapy entails fostering a collaborative approach to decision-making between healthcare providers and patients [38]. Well-informed patients play an active role in making treatment decisions, leading to better adherence and improved outcomes.

## Conclusions

The future of personalized medicine is supported by an expanding body of evidence derived from real-world studies and clinical trials. As ongoing research reveals the advantages of customized methodologies, incorporating personalized medicine will become an essential component of clinical practice. In summary, the potential of personalized medicinal therapy after stent implantation is highly promising. With the ongoing progress of technology and the advancement of our knowledge about diseases, treatment approaches will become more sophisticated and tailored to individual needs. The endeavor to achieve precise cardiovascular care will enable healthcare providers to enhance treatment outcomes, enhance the quality of life for patients, and contribute to a future in which each patient receives personalized care tailored to their genetic makeup.

## References

[1] Shen Y, Yu X, Cui J (2022). Development of biodegradable polymeric stents for the treatment of cardiovascular diseases. Biomolecules.

[2] Wu JJ, Way JA, Kritharides L, Brieger D (2019). Polymer-free versus durable polymer drug-eluting stents in patients with coronary artery disease: a meta-analysis. Ann Med Surg (Lond).

[3] Wu JJ, Way JA, Brieger D (2019). A review of the ultrathin orsiro biodegradable polymer drug-eluting stent in the treatment of coronary artery disease. Heart Int.

[4] Kim HS, Kang J, Hwang D (2021). Durable polymer versus biodegradable polymer drug-eluting stents after percutaneous coronary intervention in patients with acute coronary syndrome: the HOST-REDUCE-POLYTECH-ACS trial. Circulation.

[5] Andersen BK, Ding D, Mogensen LJH, Tu S, Holm NR, Westra J, Wijns W (2022). Predictive value of post-percutaneous coronary intervention fractional flow reserve: a systematic review and meta-analysis. Eur Heart J Qual Care Clin Outcomes.

[6] Ullah M, Wahab A, Khan SU (2023). Stent as a novel technology for coronary artery disease and their clinical manifestation. Curr Probl Cardiol.

[7] Yamaoka K, Nakagawa T, Uno T (1978). Statistical moments in pharmacokinetics. J Pharmacokinet Biopharm.

[8] Steffel J, Verhamme P, Potpara TS (2018). The 2018 European Heart Rhythm Association practical guide on the use of non-vitamin K antagonist oral anticoagulants in patients with atrial fibrillation. Eur Heart J.

[9] Urban P, Abizaid A, Chevalier B, Greene S, Meredith I, Morice MC, Pocock S (2013). Rationale and design of the LEADERS FREE trial: a randomized double-blind comparison of the BioFreedom drug-coated stent vs the Gazelle bare metal stent in patients at high bleeding risk using a short (1 month) course of dual antiplatelet therapy. Am Heart J.

[10] Elliott TL, Pfotenhauer KM (2022). Classification and diagnosis of diabetes. Prim Care.

[11] Hwang D, Koo B-K, Zhang J (2022). Prognostic implications of fractional flow reserve after coronary stenting. JAMA Netw Open.

[12] Gimbel M, Qaderdan K, Willemsen L (2020). Clopidogrel versus ticagrelor or prasugrel in patients aged 70 years or older with non-ST-elevation acute coronary syndrome (POPular AGE): the randomised, open-label, non-inferiority trial. Lancet.

[13] Pereira NL, Farkouh ME, So D (2020). Effect of genotype-guided oral P2Y12 inhibitor selection vs conventional clopidogrel therapy on ischemic outcomes after percutaneous coronary intervention. JAMA.

[14] Krucoff MW, Urban P, Tanguay JF (2020). Global approach to high bleeding risk patients with polymer-free drug-coated coronary stents: the LF II study. Circ Cardiovasc Interv.

[15] Stefanini GG, Kalesan B, Serruys PW (2011). Long-term clinical outcomes of biodegradable polymer biolimus-eluting stents versus durable polymer sirolimus-eluting stents in patients with coronary artery disease (LEADERS): 4 year follow-up of a randomised non-inferiority trial. Lancet.

[16] Meraj PM, Jauhar R, Singh A (2015). Bare metal stents versus drug eluting stents: where do we stand in 2015?. Curr Treat Options Cardiovasc Med.

[17] Windecker S, Latib A, Kedhi E (2022). Polymer-based versus polymer-free stents in high bleeding risk patients: final 2-year results from Onyx ONE. JACC Cardiovasc Interv.

[18] Jensen Lisette Okkels, Maeng M, Raungaard B (2020). Randomized comparison of the polymer-free biolimus-coated BioFreedom stent with the ultrathin strut biodegradable polymer sirolimus-eluting Orsiro stent in an all-comers population treated with percutaneous coronary intervention: the SORT OUT IX trial. Circulation.

[19] Costa RA, Abizaid A, Mehran R (2016). Polymer-free biolimus A9-coated stents in the treatment of de novo coronary lesions: 4- and 12-month angiographic follow-up and final 5-year clinical outcomes of the prospective, multicenter biofreedom FIM clinical trial. JACC Cardiovasc Interv.

[20] Carrié D, Berland J, Verheye S (2012). A multicenter randomized trial comparing amphilimus- with paclitaxel-eluting stents in de novo native coronary artery lesions. J Am Coll Cardiol.

[21] Dang Q, Li YJ, Gao L, Jin Z, Gou LX (2012). Six-month angiographic and one-year clinical outcomes of polymer free paclitaxel-eluting stent in patients with ST-segment elevation myocardial infarction: a comparison with permanent polymer sirolimus-eluting stent [Article in Chinese]. Chin Med J.

[22] Chen SL, Ye F, Zhang JJ (2013). Real polymer-free sirolimus- and probucol-eluting versus biodegradable polymer sirolimus-eluting stents for obstructive coronary artery disease: DKPLUS-Wave 1, a multicenter, randomized, prospective trial. Cardiovasc Ther.

[23] King L, Byrne RA, Mehilli J, Schömig A, Kastrati A, Pache J (2013). Five-year clinical outcomes of a polymer-free sirolimus-eluting stent versus a permanent polymer paclitaxel-eluting stent: final results of the intracoronary stenting and angiographic restenosis - test equivalence between two drug-eluting stents (ISAR-TEST) trial. Catheter Cardiovasc Interv.

[24] Byrne RA, Mehilli J, Iijima R (2009). A polymer-free dual drug-eluting stent in patients with coronary artery disease: a randomized trial vs. polymer-based drug-eluting stents. Eur Heart J.

[25] Byrne RA, Kufner S, Tiroch K (2009). Randomised trial of three rapamycin-eluting stents with different coating strategies for the reduction of coronary restenosis: 2-year follow-up results. Heart.

[26] Massberg S, Byrne RA, Kastrati A (2011). Polymer-free sirolimus- and probucol-eluting versus new generation zotarolimus-eluting stents in coronary artery disease: the intracoronary stenting and angiographic results: test efficacy of sirolimus- and probucol-eluting versus zotarolimus-eluting stents (ISAR-TEST 5) trial. Circulation.

[27] Stiermaier T, Heinz A, Schloma D (2014). Five-year clinical follow-up of a randomized comparison of a polymer-free sirolimus-eluting stent versus a polymer-based paclitaxel-eluting stent in patients with diabetes mellitus (LIPSIA Yukon trial). Catheter Cardiovasc Interv.

[28] Rozemeijer R, Stein M, Voskuil M (2019). Randomized all-comers evaluation of a permanent polymer zotarolimus-eluting stent versus a polymer-free amphilimus-eluting stent. Circulation.

[29] Romaguera R, Gómez-Hospital JA, Gomez-Lara J (2016). A randomized comparison of reservoir-based polymer-free amphilimus-eluting stents versus everolimus-eluting stents with durable polymer in patients with diabetes mellitus: the RESERVOIR clinical trial. JACC Cardiovasc Interv.

[30] Shiratori Y, Cola C, Brugaletta S (2014). Randomized comparison between polymer-free versus polymer-based paclitaxel-eluting stent: two-year final clinical results. Circ Cardiovasc Interv.

[31] Jensen LO, Maeng M, Raungaard B (2019). Comparison of the polymer-free biolimus-coated BioFreedom stent with the thin-strut biodegradable polymer sirolimus-eluting Orsiro stent in an all-comers population treated with percutaneous coronary intervention: Rationale and design of the randomized SORT OUT IX trial. Am Heart J.

[32] Zhang Y, Shen J, Li Z (2013). Two-year clinical outcomes of different drug-eluting stents with different polymer coating strategies in coronary artery heart disease: a multi-centre, randomised, controlled clinical trial. Int J Cardiol.

[33] Natsuaki M, Kozuma K, Morimoto T (2013). Biodegradable polymer biolimus-eluting stent versus durable polymer everolimus-eluting stent: a randomized, controlled, noninferiority trial. J Am Coll Cardiol.

[34] Zhang YJ, Chen F, Muramatsu T (2014). Nine-month angiographic and two-year clinical follow-up of polymer-free sirolimus-eluting stent versus durable-polymer sirolimus-eluting stent for coronary artery disease: the Nano randomized trial. Chin Med J.

[35] Windecker S, Latib A, Kedhi E (2020). Polymer-based or Polymer-free Stents in Patients at High Bleeding Risk. N Engl J Med.

[36] Ellert-Gregersen J, Jensen LO, Jakobsen L (2022). Polymer-free biolimus-coated stents versus ultrathin-strut biodegradable polymer sirolimus-eluting stents: two-year outcomes of the randomised SORT OUT IX trial. EuroIntervention.

[37] van Hemert ND, Voskuil M, Rozemeijer R (2021). 3-Year clinical outcomes after implantation of permanent-polymer versus polymer-free stent: ReCre8 landmark analysis. JACC Cardiovasc Interv.

[38] Rozemeijer R, van Muiden IG, Koudstaal S (2019). One-year clinical outcomes of patients treated with polymer-free amphilimus-eluting stents or zotarolimus-eluting stents: a propensity-score adjusted analysis. Catheter Cardiovasc Interv.

[39] Gallone G, D'Ascenzo F, Ielasi A (2022). Polymer-free biolimus-eluting stents or polymer-based zotarolimus-eluting stents for coronary bifurcation lesions. Cardiovasc Revasc Med.

[40] Koch T, Lenz T, Joner M (2021). Ten-year clinical outcomes of polymer-free versus durable polymer new-generation drug-eluting stent in patients with coronary artery disease with and without diabetes mellitus : results of the intracoronary stenting and angiographic results: test efficacy of sirolimus- and probucol- and zotarolimus-eluting stents (ISAR-TEST 5) trial. Clin Res Cardiol.

[41] Loewenstein I, Hochstadt A, Merdler I (2022). Does the use of polymer-free drug eluting stents improve clinical outcomes of patients undergoing percutaneous coronary interventions?. Coron Artery Dis.

[42] Chiarito M, Sardella G, Colombo A (2019). Safety and efficacy of polymer-free drug-eluting stents. Circ Cardiovasc Interv.

[43] Mauri L, Kereiakes DJ, Yeh RW (2014). Twelve or 30 months of dual antiplatelet therapy after drug-eluting stents. N Engl J Med.

[44] Özdemir M (2017). PIONEER AF-PCI trial [Article in Turkish]. Turk Kardiyol Dern Ars.

[45] Nogic J, Baey YW, Nerlekar N (2018). Polymer-free versus permanent polymer-coated drug eluting stents for the treatment of coronary artery disease: a meta-analysis of randomized trials. J Interv Cardiol.

[46] Verdoia M, Kedhi E, Suryapranata H, Galasso G, Dudek D, De Luca G (2020). Polymer-free vs. polymer-coated drug-eluting stents for the treatment of coronary artery disease: a meta-analysis of 16 randomized trials. Cardiovasc Revasc Med.

[47] Khatri M, Kumar S, Mahfooz K (2023). Clinical outcomes of polymer-free versus polymer-coated drug-eluting stents in patients with coronary artery disease: a systematic review and meta-analysis. Cureus.

[48] Ondondo BO (2018). Platelet function testing for cardiac surgery patients on antiplatelet therapy: the extreme variability of point-of-care tests. Biomed Pharmacol J.

[49] Siton O, Bernheim-Groswasser A (2014). Reconstitution of actin-based motility by vasodilator-stimulated phosphoprotein (VASP) depends on the recruitment of F-actin seeds from the solution produced by cofilin. J Biol Chem.

